# Estimation of Interference Arrival Direction Based on a Novel Space-Time Conversion MUSIC Algorithm for GNSS Receivers

**DOI:** 10.3390/s19112570

**Published:** 2019-06-05

**Authors:** Hao Wang, Qing Chang, Yong Xu, Xianxu Li

**Affiliations:** 1 School of Electronic and Information Engineering, Beihang University, No. 37 Xueyuan Road, Haidian District, Beijing 100191, China; wanghao1989@buaa.edu.cn; 2 Institute of Unmanned Systems Research, Beihang University, No. 37 Xueyuan Road, Haidian District, Beijing 100191, China; 3 State Grid Information and Telecommunication Branch, Beijing 100761, China; lixianxu@buaa.edu.cn

**Keywords:** DOA, MUSIC, focusing parameter, subspace, space-time

## Abstract

In the estimation of the direction of arrival (DOA) for interference signals, the direction-finding error of the multiple signal classification (MUSIC) algorithm will increase in the case of multiple interferences or when the interfering signal power is weak. In this paper, a space-time conversion MUSIC (STC-MUSIC) algorithm is proposed, and the concept of a focusing parameter is introduced to improve the performance of the DOA estimation. Meanwhile, a method of variable step size peak search is proposed to reduce the amount of calculation of the STC-MUSIC algorithm. The final simulation and experimental results show that the STC-MUSIC algorithm improves the purity of the noise subspace effectively, thus improving the precision and robustness of the DOA estimation for interference signals significantly. In comparison to traditional algorithms, the convergence, stability, root mean square error (RMSE) and other performance characteristics are improved greatly.

## 1. Introduction

With the complications of the electromagnetic environment, the global navigation satellite system (GNSS) faces increasing unintentional or intentional interference. For the navigation terminal, in addition to the stable anti-interference ability, it also needs to have the ability to estimate the interference direction, so as to provide the necessary conditions for destroying the interference source accurately in the military environment [1].

The general technique for interference source localization usually needs to estimate the direction of arrival (DOA) for the interference signal to obtain azimuth and elevation angles [2,3]. The multiple signal classification (MUSIC) algorithm is a common DOA estimation algorithm based on the array that can measure multiple signals simultaneously and eliminate limitations, due to the beam width of the array [4]. Its performance is better than the Capon algorithm and directional antenna, phase interferometer and other methods [5].The shortcomings of the traditional MUSIC algorithm primarily include (1) the DOA estimation of the coherent signal cannot be obtained accurately; (2) the number of received signals is limited by the number of array elements, and needs to be smaller than the number of array elements; (3) as the jamming-to-noise ratio (JNR) decreases, the DOA estimation error will increase; and (4) DOA estimation accuracy is sensitive to the characteristics of the RF channel and mutual coupling [6,7]. For deficiencies in the case of coherent signals, The spatial smoothing algorithm has been used in References [8] and [9]. This method divides the array into several sub-arrays, which overlap each other, and obtains the mean of all sub-array covariance matrices. The covariance matrix is restored to a full rank state to ensure that the constructed signal subspace and the noise subspaces are orthogonal. Similarly, the authors in Reference [10] used a differential smoothing method to obtain accurate DOA estimation for coherent signals. To address the limitation in the number of array elements in the algorithm, a DOA estimation method based on array interpolation was proposed in Reference [11] to increase the number of resolvable sources. In Reference [12], a vectorized MUSIC algorithm was introduced, which optimizes the array by constructing a differential summation to obtain more degrees of freedom. A coprime array interpolation approach was proposed in References [13] and [14] to improve the number of achievable degrees-of-freedom. In Reference [15], a fast gridless maximum likelihood method was proposed, which improved computational efficiency effectively and can detect more sources. To improve the accuracy for direction-finding, an extended-aperture DOA estimation algorithm based on a unitary root-MUSIC algorithm for a coprime array was proposed in Reference [16]. Through simulation and comparison, the proposed method can obtain more accurate direction-finding results. In Reference [17], a novel two-stage processing method was proposed that can effectively decompose a signal into a mixture of correlated and uncorrelated components, thereby improving the accuracy of the algorithm. In practice, arrays sensitive to polarization can also be used to extend the characteristic information from the received signal to improve the direction-finding accuracy and the degree of freedom of the array [18]. In addition, sparse representation or compressed sensing can be used to obtain better performance [19], but the computational complexity is increased significantly. In References [20] and [21], a novel estimation method was proposed for obtaining the sample covariance matrix of a signal. The method effectively reduces the computational complexity and improves the performance of the algorithm in the case of a limited sample length.

A space-time conversion MUSIC (STC-MUSIC) algorithm is proposed in this paper, which effectively expands the order of the signal covariance matrix, improves the isolation of the target signal subspace and the noise subspace, and thus obtains a purer noise subspace that improves the spectral function accuracy and robustness of the traditional algorithm. Especially in the multi-interference scenario, the performance is improved significantly compared to the traditional method. At the same time, a direction-finding focusing parameter is introduced, and the eigenvectors of the noise subspace can be adjusted to obtain optimal DOA estimation performance. Finally, the direction-finding errors for different JNR conditions are evaluated.

The outline for the remainder of this paper is as follows. Section 2 presents the signal model. The underlying theory and signal process for the STC-MUSIC algorithm and the focusing parameter are presented in Section 3. Simulation results and conclusions are presented in Section 4 and Section 5, respectively.

## 2. Signal Model

**Figure 1:**
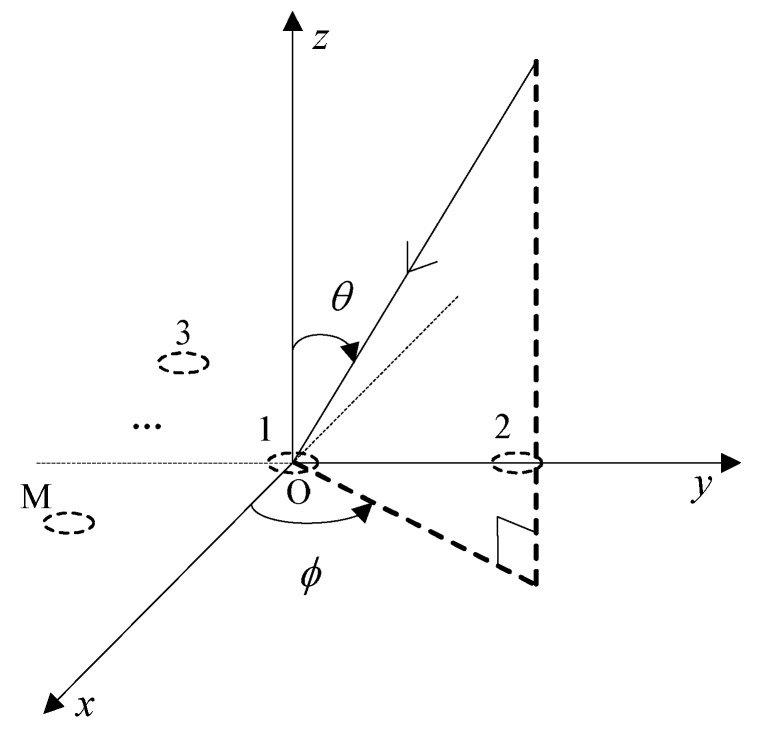
Schematic diagram of the array.

We consider an arbitrary array with M elements. Each array element has the same directional characteristics, and O is the center of the array, as shown in Figure 1.

It is assumed that the P satellite signals and the Q interference signals are incident on the array and satisfy the condition of far field incidence. (The far field means that the interference source is far enough away from the array that the wave reaching the array can be regarded as a parallel wave. The actual interference signal generally satisfies this condition). The signal received by the array can be expressed as

(1) $$x(t)=\sum_{p=1}^{P}a(\theta_{p},\varphi_{p})s_{p}(t)+\sum_{q=1}^{Q}a(\theta_{q},\varphi_{q}j_{q}(t)+n(t),$$

where $x(t)={\left[x_{1}(t),x_{2}(t),\cdot \cdot \cdot ,x_{M}(t)\right]}^{T}$ represents the signal obtained by the M dimensional array at the unit sampling time; $s_{p}(t)$ and $j_{q}(t)$ represent the envelopes for the corresponding useful signals and interference signals, respectively; P and Q represent the number of satellite signals and interference signals, respectively; and n(t) represents the noise vector received by the M dimensional array. The vectors $a(\theta_{p},\varphi_{p})$ and $a(\theta_{q},\varphi_{q})$ represent the steering vectors corresponding to the useful signal and interference signal, respectively, where θ represents the elevation angle and φ represents the azimuth angle.

(2) $$a(\theta ,\varphi )={\left[e^{-ju^{T}p_{1}},e^{-ju^{T}p_{2}},\cdots ,e^{-ju^{T}p_{M}}\right]}^{T},$$

where

(3) $$u=\frac{2\pi }{\lambda_{s}}{\left[\begin{array}{ccc} \sin\theta \cos\varphi & \sin\theta \sin\varphi & \cos\theta \end{array}\right]}^{T},$$

and $\lambda_{s}$ represents the incident signal wavelength, and

(4) $$p_{k}={\left[\begin{array}{ccc} p_{xk} & p_{yk} & p_{zk} \end{array}\right]}^{T},k=1,2,\cdots M,$$

$p_{k}$ represents the three-dimensional coordinates of the array element.

It should be noted that regarding the DOA estimation based on the array antenna, the distinction between the narrowband and the wideband signals is relative to the array. In general, we require that the maximum time difference between the signals arriving at each element is small enough, i.e., the signal envelope received by each element is consistent. We assume that the maximum time difference is $\tau_{\max}$, and the signal bandwidth is $B_{w}$, then it needs to satisfy

(5) $$\tau_{\max}\ll \frac{1}{B_{w}},$$

i.e., the maximum time difference between the signals arriving at each element is much smaller than the equivalent time width of the signal. If so, we call the array as a coherent array with respect to the signal, corresponding to a narrowband signal, otherwise it is a wideband signal.

$\tau_{\max}$ is related to the total aperture of the array antenna. i.e.,

(6) $$\tau_{\max}=\frac{D}{c}=\frac{D}{\lambda_{s}f},$$

where *D* represents the total aperture of the array antenna, c represents the speed of light and *f* represents the signal frequency. In general, the element spacing of most array antennas is designed to be about $\lambda_{s}/2$. In addition, the size and shape of the array also need to be considered for miniaturization in applications, so *D* and $\lambda_{s}$ are basically on the same order of magnitude. Substitute (6) into (5), we can compare *f* and $B_{w}$ to determine whether the signal is narrowband or wideband. Since the GNSS signal is generally in the L-band, the signal bandwidth is generally 2 MHz to 20 MHz, and the interference bandwidth for the GNSS signal is also in this range. Therefore, for the GNSS receiver, the interference signal generally faced is basically a narrow band. Of course, broadband interference may also exist in practice, but this paper does not focus on this research.

## 3. STC-MUSIC Algorithm

### 3.1. Principle and Signal Processing Process

First, if the number of elements is *M*, and the number of snapshots is *N*, then the received signal by the array for one data block can be expressed as

(7) $$X=\left[\begin{array}{ccccc} x_{11} & x_{12} & \cdots & x_{1(N-1)} & x_{1N}\\ x_{21} & x_{22} & \cdots & x_{2(N-1)} & x_{2N}\\ \vdots & \vdots & \cdots & \vdots & \vdots \\ x_{M1} & x_{M2} & \cdots & x_{M(N-1)} & x_{MN} \end{array}\right]$$

The STC-MUSIC algorithm is built under the space-time structure.

**Figure 2:**
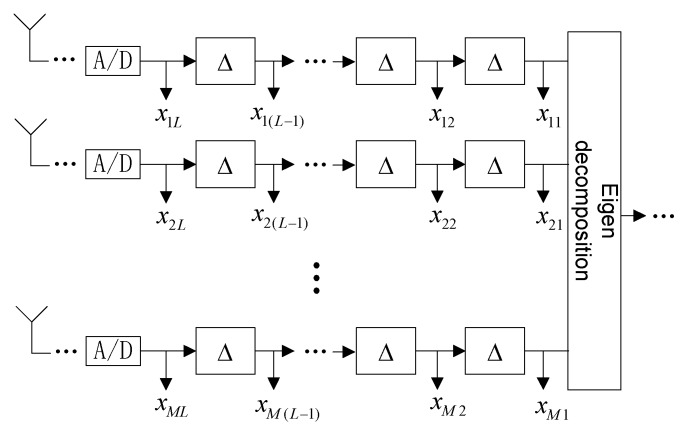
STC-MUSIC algorithm structure diagram.

As shown in Figure 2, for the processing of the space-time structure, there are (L−1) delay units after each array element, and the delay is Δ. After the space-time domain conversion of X, we obtain

(8) $$X_{ST}=\left[\begin{array}{ccccc} x_{1L} & x_{1(L+1)} & x_{1(L+2)} & \cdots & x_{1N}\\ \vdots & \vdots & \vdots & \cdots & \vdots \\ x_{11} & x_{12} & x_{13} & \cdots & x_{1(N-L+1)}\\ x_{2L} & x_{2(L+1)} & x_{2(L+2)} & \cdots & x_{2N}\\ \vdots & \vdots & \vdots & \cdots & \vdots \\ x_{21} & x_{22} & x_{23} & \cdots & x_{2(N-L+1)}\\ \begin{array}{l} \vdots \\ \vdots \end{array} & \begin{array}{l} \vdots \\ \vdots \end{array} & \begin{array}{l} \vdots \\ \vdots \end{array} & \begin{array}{l} \cdots \\ \cdots \end{array} & \begin{array}{l} \vdots \\ \vdots \end{array}\\ x_{ML} & x_{M(L+1)} & x_{M(L+2)} & \cdots & x_{MN}\\ \vdots & \vdots & \vdots & \cdots & \vdots \\ x_{M1} & x_{M2} & x_{M3} & \cdots & x_{M(N-L+1)} \end{array}\right]$$

Then, we calculate the covariance matrix of $X_{ST}$,

(9) $$R_{ST}=E[X_{ST}X_{\mathrm{ST}}{}^{H}]$$

In practice, we usually use the sampling covariance matrix, denoted by ${\hat{R}}_{ST}$, and the matrix dimension is represented by ML×ML. After the eigen decomposition of ${\hat{R}}_{ST}$, we yield

(10) $${\hat{R}}_{ST}=\sum_{i=1}^{ML}\lambda_{i}v_{i}v_{i}^{H}={\hat{U}}_{j}\Lambda_{j}{\hat{U}}_{j}^{H}+{\hat{U}}_{n}\Lambda_{n}{\hat{U}}_{n}^{H},$$

where $\lambda_{i}$ represents the eigenvalue [22].

(11) $$\Lambda_{j}=\left[\begin{array}{cccc} \lambda_{1} & & & \\ & \lambda_{2} & & \\ & & \ddots & \\ & & & \lambda_{K} \end{array}\right],$$

represents a diagonal array containing K large eigenvalues. ${\hat{U}}_{j}=[v_{1},v_{2},\cdots ,v_{K}]$ represents an interference signal subspace formed by the eigenvectors corresponding to K large eigenvalues.

(12) $$\Lambda_{n}=\left[\begin{array}{cccc} \lambda_{K+1} & & & \\ & \lambda_{K+2} & & \\ & & \ddots & \\ & & & \lambda_{M\times L} \end{array}\right],$$

represents a diagonal array of M×L−K small eigenvalues. ${\hat{U}}_{n}=[v_{K+1},v_{K+2},\cdots ,v_{M\times L}]$ represents the noise subspace formed by the eigenvectors corresponding to the M×L−K eigenvalues [23]. Since the GNSS uses the spread spectrum technology, the satellite signal is embedded in the white noise, and the signal power is about 20 dB–30 dB lower than the noise power, and the influence of satellite signals on the construction of noise subspace and interference signal subspace can be ignored.

The spectral function of the STC-MUSIC algorithm is

(13) $$P_{s}(\theta ,\varphi )=\frac{1}{a_{st}^{H}(\theta ,\varphi ){\hat{U}}_{n}{\hat{U}}_{n}^{H}a_{st}(\theta ,\varphi )},$$

where $a_{st}(\theta ,\varphi )$ represents the space-time steering vector,

(14) $$a_{st}(\theta ,\varphi )=a(\theta ,\varphi )⊗a_{t},$$

a(θ,φ) as shown in (2), represents the space steering vector, and ⊗ represents the Kronecker product.

(15) $$a_{t}=(1,e^{-j2\pi (f_{I}+f_{D})\Delta t},\cdots ,e^{-j2\pi (L-1)(f_{I}+f_{D})\Delta t}),$$

is the delay vector of the received signal. In the delay vector, Δt represents the sampling period, $f_{I}$ represents the intermediate frequency of the GNSS receiver after down-conversion, $f_{D}$ represents the Doppler frequency, and (L−1) represents the number of delaying units. Based on Equation (13), we traverse (θ,φ) and perform a two-dimensional search to find the peak of the spectral function, and then make a decision to determine the direction of the interference signal.

Since the array signal is delayed, the obtained covariance matrix includes not only the correlation items between the array elements at the current time, but also the correlation items between the array elements at different moments in the past. The signal changes from a simple spatial domain to a space-time two-dimensional domain. Therefore, the accuracy of the noise subspace and the interference subspace will be improved when performing eigen decomposition.

### 3.2. Variable Step Size Peak Search

Due to the introduction of the space-time conversion, the eigenvector dimension corresponding to the noise subspace and the dimension of the space-time steering vector are all expanded. Therefore, the spectral peak search will bring more computation than before. We introduce a variable step size peak search method to reduce the computational complexity of the STC-MUSIC algorithm.

We assume that the number of interferences is *Q*. When searching for spectral peaks, we first search by a larger step size ΔH. In order to ensure the convergence of the algorithm, we generally can’t take too large values. In this paper, we take 1° or 2°, and then we can get *Q* extreme values, their corresponding angles can be expressed as

(16) $$\{(\theta_{1},\varphi_{1}),(\theta_{2},\varphi_{2}),\ldots ,(\theta_{Q},\varphi_{Q})\},$$

and the corresponding two-dimensional search times are

(17) St_1=(1+90°/ΔH)⋅(1+180°/ΔH).

Then we can search in a small step size $\Delta h_{1}$, which can take 0.1°, the search intervals for the *Q* interferences are

(18) $$\{(\theta_{1}\pm \Delta \theta ,\varphi_{1}\pm \Delta \varphi ),(\theta_{2}\pm \Delta \theta ,\varphi_{2}\pm \Delta \varphi ),\ldots ,(\theta_{Q}\pm \Delta \theta ,\varphi_{Q}\pm \Delta \varphi )\}.$$

In order not to miss the peak as much as possible, the value of Δθ and Δφ cannot be less than ΔH/2. The corresponding two-dimensional search times are

(19) $$St\_2=Q\cdot (1+2\Delta \theta /\Delta h_{1})\cdot (1+2\Delta \varphi /\Delta h_{1}).$$

The total search times are the sum of Equations (17) and (19). Of course, according to actual needs, we can follow the above steps to search again with a smaller step size $\Delta h_{2}$.

We take three interferences as an example to calculate. It takes 901×1801 times to search globally with a step of 0.1°, and 91×181+11×11×3 times with a variable step of 1° and 0.1°. Comparing the two methods, the number of searches has been reduced by 1605867 times, which is about a hundred times lower.

### 3.3. Direction-finding Focusing Parameter

In the eigen decomposition, the eigenvectors corresponding to the larger eigenvalues are formed into the subspace of the interference signal, and the eigenvectors corresponding to the smaller eigenvalues are formed into the noise subspace. Due to the inevitable crossover leakage between the interference signal subspace and the noise subspace, to obtain a purer noise subspace, the eigenvector corresponding to the smaller eigenvalue can be selected to constitute the noise subspace. The STC-MUSIC algorithm can construct a higher-dimensional eigenmatrix, which provides a greater choice space for obtaining a purer noise subspace. Therefore, in the STC-MUSIC algorithm, the boundary value of the eigenvalue has a larger selection range, and the boundary value also affects the final direction-finding accuracy. We use K to represent the boundary value of the eigenvalues in descending order, and 1≤K<M×L. In this range, the value of *K* can be larger than the traditional method, and the eigenvector corresponding to the smaller eigenvalue can be selected to obtain a purer noise subspace, thereby improving the accuracy of the spectral function. K usually has an optimal interval, within a certain range, the larger the value, the higher the direction-finding accuracy, but it is not the larger the better. When K is close to the maximum, i.e., K=M×L−1, only one eigenvector can be used to construct the noise subspace. Thus, the accuracy of the spectral function may decrease. To facilitate an intuitive understanding, we introduce the concept of a direction-finding focusing parameter to define K visually, which we call the focusing parameter. The STC-MUSIC algorithm provides us with a way to improve the performance of the DOA estimation by changing the focusing parameter, and it is more prominent in multiple interference situations.

Next, we are concerned with how to obtain the optimal focusing parameter. Since the noise usually does not exhibit autocorrelation and the features are stable, after the eigen decomposition of the covariance matrix, the eigenvalues corresponding to the noise subspace are very small and the sizes are basically the same, so we can use the following method to determine *K*. First, we arrange the *ML* eigenvalues in descending order, and define

(20) $$\delta_{i}=\mathrm{lg}(\frac{\lambda_{i}}{\lambda_{i+1}}),$$

with i=1,2,⋯M×L−1, where $\delta_{i}$ can be called a discriminating factor. We calculate the $\delta_{i}$ in order. Since the size of the small eigenvalues corresponding to the noise subspace is almost the same, after the transition from the large eigenvalue to the small eigenvalue, when $\lambda_{i}/\lambda_{i+1}\approx 1$ and $\delta_{i}$ tends to 0, the corresponding value of i−1 is the boundary value, i.e., K=i−1 can be used as the optimal focusing parameter. In practice, the threshold of $\delta_{i}$ can be set to $10^{-2}$. In the next simulation, we will combine the actual data to verify the above conclusions.

### 3.4. Delay Units

In the STC-MUSIC algorithm, we also need to determine the number of delay units, i.e., the range of L, which requires a comprehensive consideration of the calculated amount and the final performance for direction-finding. Generally, we can achieve stable performance by taking L=2∼5. Continuing to increase L will result in more calculations, and the DOA estimation performance will no longer increase significantly. In addition, if *L* is too large, it may cause distortion to the signal. 

## 4. Simulation Results and Analysis

**Figure 3:**
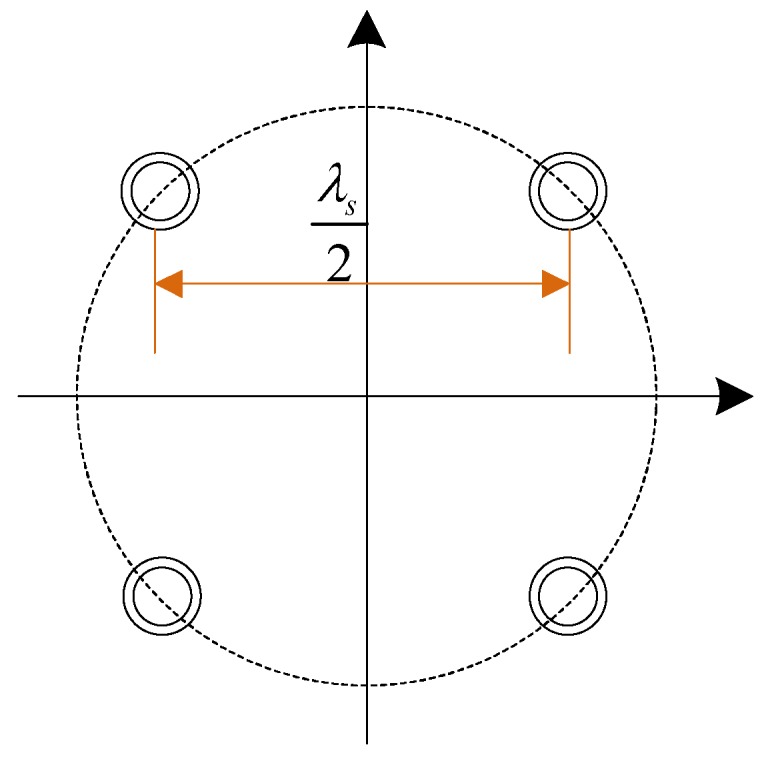
The antenna array configuration. ($\lambda_{s}$ represents the signal wavelength).

In this section, we simulate and analyze the STC-MUSIC algorithm to verify and evaluate the performance of the algorithm. First, it is necessary to simulate the influence of the data block length (i.e., the number of snapshots) on the STC-MUSIC algorithm to obtain the optimal interference direction-finding scheme. Second, compared with the traditional MUSIC algorithm, the accuracy and robustness of the DOA estimation by the STC-MUSIC algorithm under single interference and multiple interference conditions are verified, and then the method for determining the optimal focusing parameter is verified and analyzed. Finally, the direction-finding error under different JNRs are evaluated to further verify the performance of the STC-MUSIC algorithm. In the following simulations, the number of the array elements is 4, the antenna array configuration is shown in Figure 3, and the array element spacing is a half wavelength. The basic simulation parameters are shown in Table 1.

### 4.1. Simulation of Data Block Length

**Figure 4:**
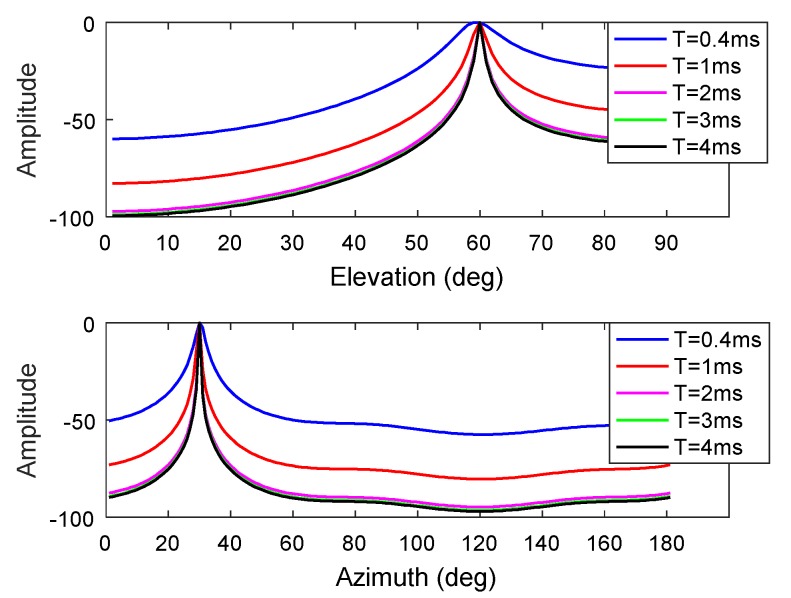
Estimation results for elevation and azimuthal angles obtained under different data block length conditions. (Top: The corresponding elevation angle spectrum function curves when the azimuthal angle is 30°; Bottom: The corresponding azimuthal angle spectrum function curves when the elevation angle is 60°).

In practice, we usually process the received signal in the form of data blocks. The length of the data block affects the convergence and accuracy of the algorithm, but we also need to consider the computational complexity. The simulation parameters are as follows: A Gaussian interference with a JNR of 0 dB, an elevation angle of 60°, and an azimuth angle of 30°, the bandwidth is about 2 MHz. We then alter the length of the data block processed each time and estimate the DOA of the interference, and the number of delay units is set to 3. As shown in Figure 4, the range of T is between 0.4 ms and 4 ms. When T=0.4 ms, the algorithm tends to converge, and the direction-finding result is accurate. When T=1 ms, the spectral functions of the azimuth angle and elevation angle are steeper at the interference angle. With further increases in T, the slope of the spectral function at the peak is essentially stable, and there is no obvious change, but the amount of calculation is gradually increasing. Based on the real-time performance, we can set the time length for the data block to 1 ms.

### 4.2. Analysis of the Optimal Focusing Parameter

In Section 3, we introduce a method for calculating the optimal focusing parameter, which uses the feature that the small eigenvalues are almost equal. We first verify the method and then discuss the influence of the focusing parameter on the final direction-finding accuracy.

First, we verify the distribution law of the eigenvalues after the eigen decomposition of the covariance matrix in the STC-MUSIC algorithm. We set two interference scenarios, which are single interference and two interferences. The specific parameters are shown in Table 2. The number of delay units of the STC-MUSIC algorithm is set to 3, i.e., *L* = 4.

**Figure 5:**
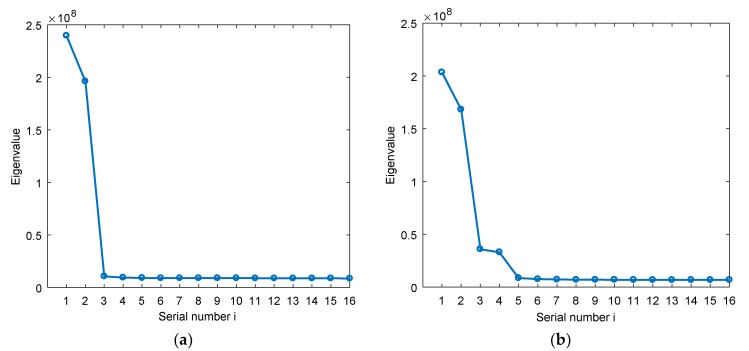
The distribution of the eigenvalues. (**a**) The distribution of the eigenvalues in scene 1; (**b**) The distribution of the eigenvalues in scene 2.

We arrange the eigenvalues after eigen decomposition in each interference scene in descending order, Figure 5a,b show the distribution of the eigenvalues corresponding to Scene 1 and Scene 2, respectively. It can be seen that the small eigenvalues after large eigenvalues in each scene are basically the same, and there is no significant change between them, so that we can get the optimal focusing parameter according to Equation (20).

Next, we discuss the influence of the focusing parameter on the accuracy of DOA estimation. The simulation parameters are set as follows: A single-carrier interference with an elevation angle of 20° and an azimuth angle of 90°; a Gaussian interference with an elevation angle of 45° and an azimuth angle of 120°; a BPSK modulated interference with an elevation angle of 60° and an azimuth angle of 30°. The JNR is 0 dB, and the bandwidth of the Gaussian interference and BPSK modulated interference is about 2 MHz. The number of delay units of the STC-MUSIC algorithm is 3, i.e., L=4.

**Figure 6:**
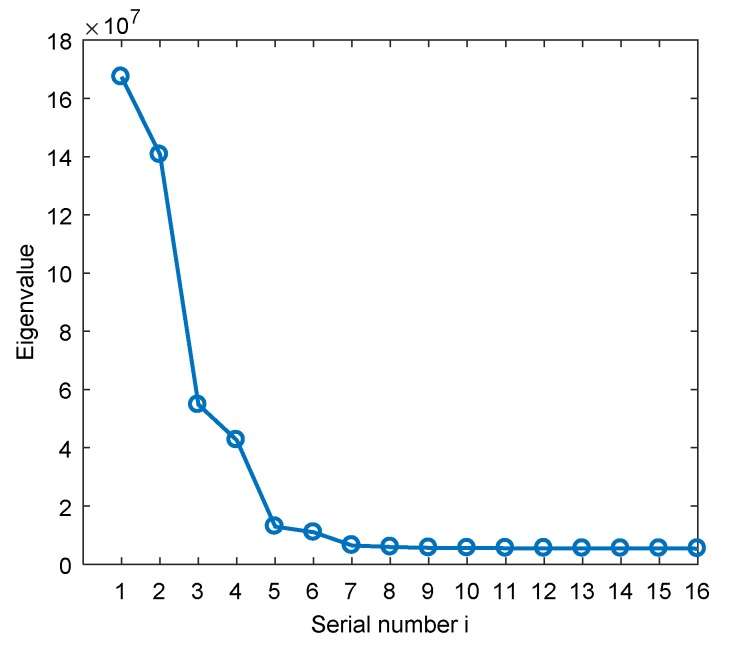
The distribution of the eigenvalues.

**Figure 7:**
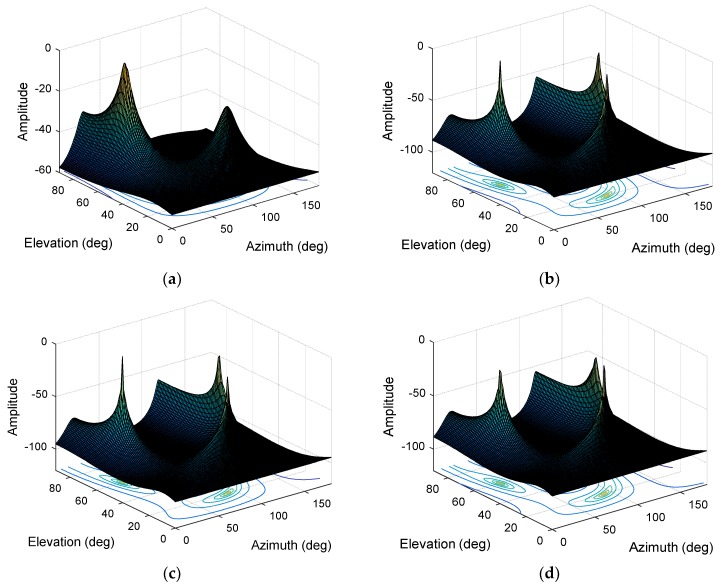
The normalized spectral function. (**a**) The normalized spectral function at K=4; (**b**) The normalized spectral function at K=8; (**c**) The normalized spectral function at K=12; (**d**) The normalized spectral function at K=15.

As shown in Figure 6, the corresponding eigenvalues after the eigendecomposition of the sampling covariance matrix are obtained. We find that, after transitioning from large eigenvalues to small eigenvalues, the small eigenvalues are essentially the same and almost unchanged. Calculated by Equation (20), we can get $\delta_{9}\leq 0.01$. The focusing parameter thus enters the optimal interval when K=8. We analyze and verify the direction-finding results with different focusing parameters. As shown in Figure 7a, when K=4, the spectral function does not form the correct peak in the interference direction. As we continue to increase K, as shown in Figure 7b,c, we obtain the spectral functions of K=8 and K=12. The direction-finding results are accurate and stable and it can be seen that there is no significant difference between the two spectral functions. When K continues to increase to the maximum allowable value i.e., K=15. As shown in Figure 7d, the peak value and accuracy of the spectral function decrease. In summary, the simulation results are consistent with the analysis results.

**Figure 8:**
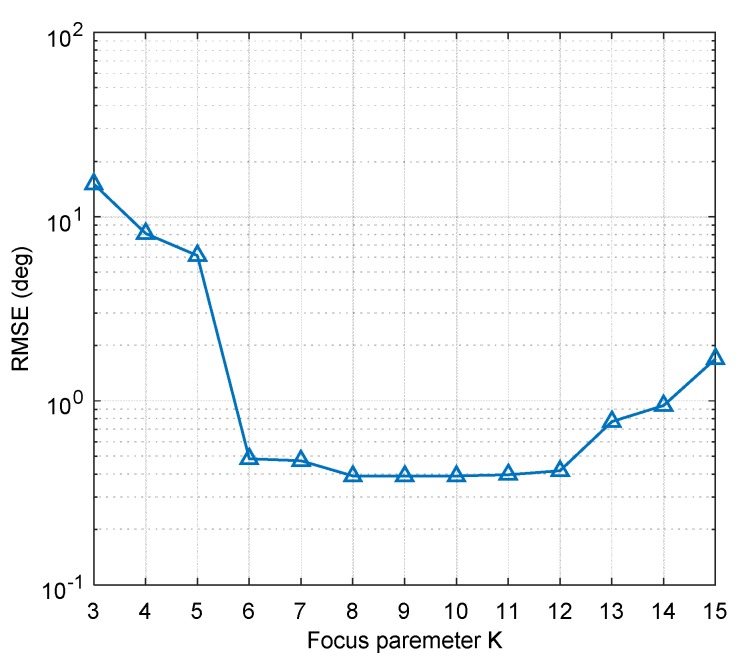
The direction-finding RMSE of STC-MUSIC algorithm with different focusing parameters.

We also calculated the root mean square error (RMSE) of the direction-finding results with different focusing parameters. As shown in Figure 8, it is the result of 100 Monte Carlo experiments, and the peak search steps are 1° and 0.1°. We can conclude that the optimal interval is 8≤K≤12. In the interval of K<6, the algorithm does not converge, and the direction of the three interferences cannot be obtained accurately. In practice, we do not need to find an interval and can directly calculate the boundary value of the optimal interval as the best focusing parameter according to the proposed method.

### 4.3. Performance Verification of the STC-MUSIC Algorithm

#### 4.3.1. Single Interference Scenario

**Figure 9:**
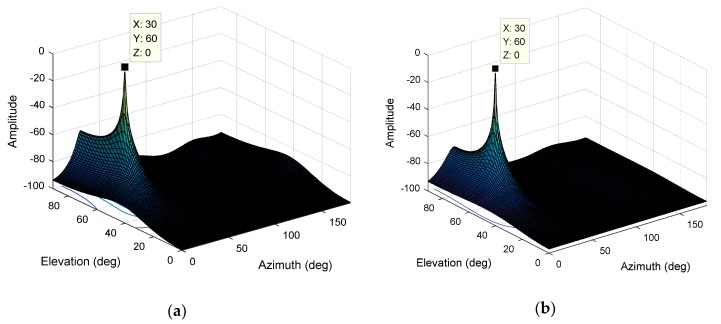
The normalized spectral function. (**a**) The normalized spectral function derived from the MUSIC algorithm (M=4, K=2); (**b**) The normalized spectral function derived from the STC-MUSIC algorithm (M=4,L=4,K=2).

**Figure 10:**
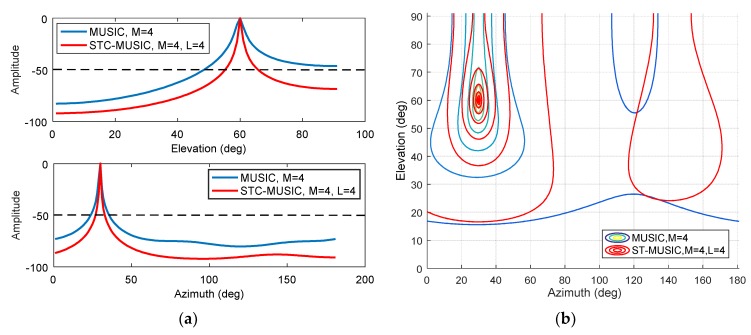
(**a**) Estimation results for elevation and azimuth obtained by MUSIC and STC-MUSIC algorithms (Top: The corresponding elevation angle spectrum function curves when the azimuthal angle is 30°; Bottom: The corresponding azimuthal angle spectrum function curves when the elevation angle is 60°); (**b**) Contour map of the spectral function.

The interference is set to a Gaussian interference with a JNR of 0 dB, an elevation angle of 60°, and an azimuth angle of 30°, the bandwidth is about 2MHz. The number of delay units in the STC-MUSIC algorithm is set to 3, i.e., L=4, and the focusing parameter is calculated according to Equation (20) to obtain K=2, and the value of *K* corresponding to the MUSIC algorithm is also 2. As shown in Figure 9a,b, we give the three-dimensional normalized spectral function graph obtained by the MUSIC algorithm and the STC-MUSIC algorithm, respectively. Both methods estimate the direction of the interference signal accurately. We further analyze and draw the spectral function curves corresponding to the azimuth and elevation angles, respectively. As shown in Figure 10a, the spectral function curve obtained using the STC-MUSIC algorithm is steeper at the interference angle. In practice, the direction finding result we sometimes give is a range. Obviously, when a threshold is set for the spectral function, the range of the angle obtained by the STC-MUSIC algorithm is narrower, so the convergence is better relatively. Figure 10b shows the contour map of the spectral function. The abscissa is the azimuth angle, and the ordinate is the elevation angle. The figure clearly shows the differences in the convergence between the two algorithms from another angle, and the direction-finding result obtained by the STC-MUSIC algorithm is more accurate.

#### 4.3.2. Multiple Interference Scenario

**Figure 11:**
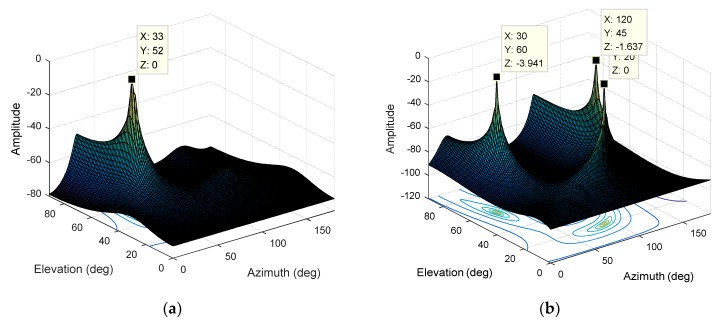
(**a**) The normalized spectral function derived from the MUSIC algorithm (M=4, K=3); (**b**) The normalized spectral function derived from the STC-MUSIC algorithm (M=4,L=4,K=8).

The interference parameters are set as follows: A single-carrier interference with an elevation angle of 20° and an azimuth angle of 90°, a Gaussian interference with an elevation angle of 45° and an azimuth angle of 120°, and a BPSK modulated interference with an elevation angle of 60° and an azimuth angle of 30°. The JNR is 0 dB, and the bandwidth of the Gaussian interference and BPSK modulated interference is about 2 MHz. We first use the MUSIC algorithm for direction finding, and *K* can only take 3. As shown in Figure 11a, for the case of multiple interferences, the spectral function derived from the traditional MUSIC algorithm based on four array elements is completely distorted, and it is impossible to estimate the direction of the three interferences. There is only one peak in the spectral function and the error is large.

Then, we use the STC-MUSIC algorithm for direction-finding. For the case of multiple interferences, the cross-diffusion between the interference subspace and the noise subspace will be aggravated. The advantage of using the focusing parameter in the STC-MUSIC algorithm is more evident. Due to the expansion of the covariance matrix, we can select the eigenvectors corresponding to the smaller eigenvalues to construct a purer noise subspace, which increases the isolation between the interference subspace and the noise subspace. Then, we calculate the focusing parameter according to Equation (20) to get K=8. As shown in Figure 11b, the spectral function forms obvious peaks in the directions corresponding to the three interferences and the DOA estimation results are accurate.

### 4.4. Evaluation of DOA Estimation Error

In this section, the performance of the STC-MUSIC algorithm is evaluated mainly from the aspect of the direction-finding error. Through 100 Monte Carlo experiments, we calculate and record the RMSE of the DOA estimation result, and the peak search steps are 1° and 0.01°.

(21) $$\mathrm{RMSE}=\sqrt{\frac{1}{100Q}\sum_{n}^{100}\sum_{q}^{Q}\left[{\left({\hat{\theta }}_{q}^{\left(n\right)}-\theta_{q}\right)}^{2}+{\left({\hat{\varphi }}_{q}^{\left(n\right)}-\varphi_{q}\right)}^{2}\right]},$$

where $\theta_{q}$ and $\varphi_{q}$ are the set elevation angle and azimuth angle, respectively, and ${\hat{\theta }}_{q}^{\left(n\right)}$ and ${\hat{\varphi }}_{q}^{\left(n\right)}$ are the corresponding estimates of the nth trial. *Q* is the number of interference signals.

**Figure 12:**
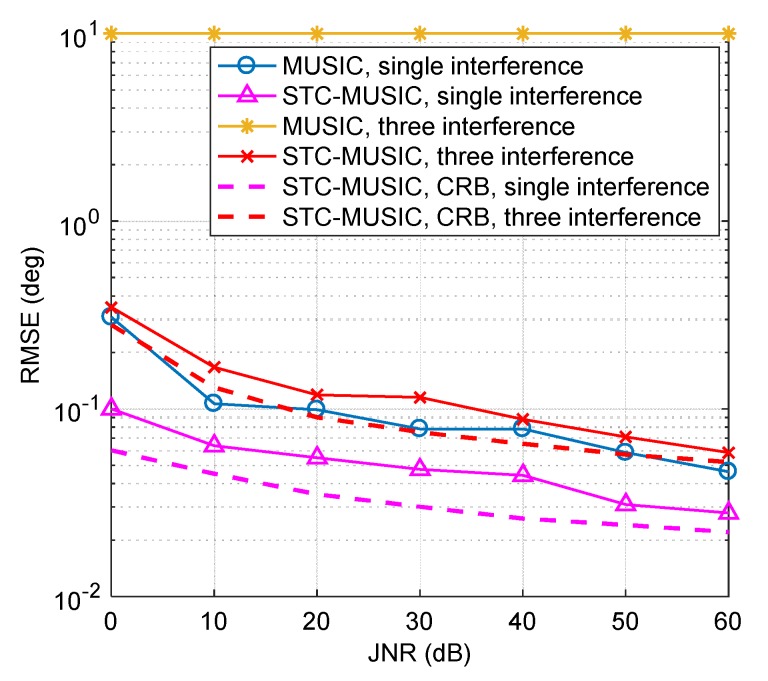
The direction-finding RMSEs of the MUSIC algorithm and STC-MUSIC algorithm under different JNR cases.

We simulate two interference scenarios: Single interference and three interferences. The JNR range is set to 0 dB–60 dB, with an interval of 10 dB. The interference direction is estimated using the MUSIC algorithm and the STC-MUSIC algorithm, and the corresponding RMSE is calculated. We also give the Cramer-Rao bound (CRB) of the STC-MUSIC algorithm in the corresponding scene. As shown in Figure 12, as the interference intensity increases, the direction-finding errors of the two algorithms gradually decrease. In the case of single interference, the STC-MUSIC algorithm is significantly better than the traditional MUSIC algorithm, and the direction-finding error is smaller. For the case of three interferences, the traditional MUSIC algorithm cannot converge. However, the STC-MUSIC algorithm can still get the direction-finding result normally, and the RMSE remains low. In summary, the simulation results verify the performance and advantages of the STC-MUSIC algorithm, which effectively improves the accuracy and robustness of the traditional DOA estimation algorithm. In addition, the STC-MUSIC algorithm maintains high precision when the interference signal is weak.

### 4.5. Test based on Actual Signal

**Figure 13:**
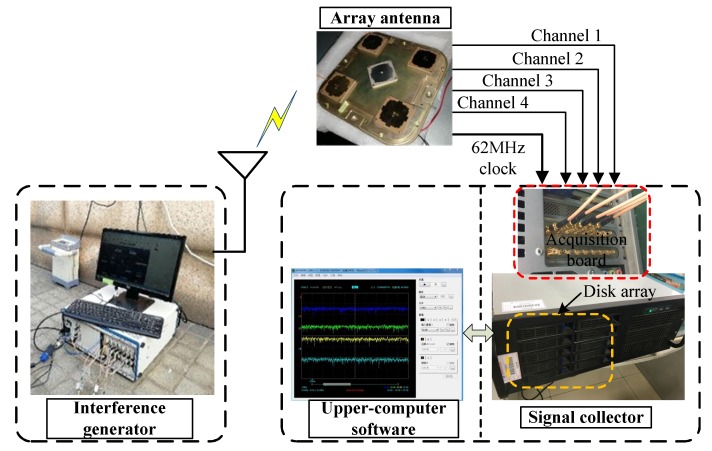
Schematic diagram of signal acquisition scheme.

We used a 4-element array antenna to acquire the signal. As shown in Figure 13, the RF frequency is 1268.52 MHz and the array element spacing is half wavelength. Each array element contains a down conversion channel and outputs an intermediate frequency analog signal, respectively. The intermediate frequency is 46.52MHz, and signal bandwidth is about 20MHz. The middle element is irrelevant to this test, and the channel is closed. For the signal collector, we used the AlazarTech ATS 9440, a four-channel acquisition card. The A/D is 14 bits and the sampling rate is set to 62 MHz. The interference generator includes two RF channels and supports the simultaneous output of two different interferences, the interference parameters are shown in Table 3. 

**Figure 14:**
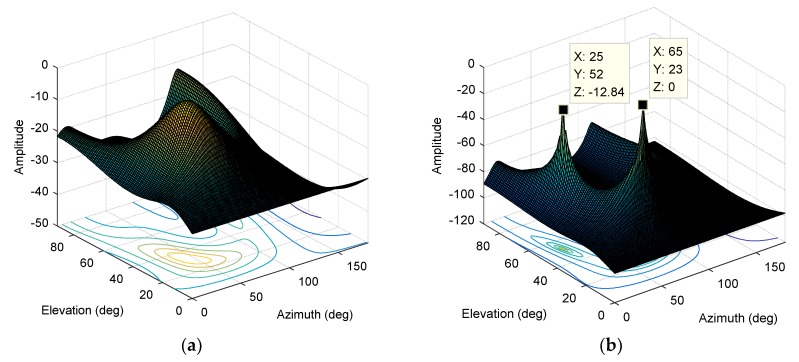
(**a**) The normalized spectral function derived from the MUSIC algorithm; (**b**) The normalized spectral function derived from the STC-MUSIC algorithm (L=4, K=12).

The results of the DOA estimation of the MUSIC algorithm and the STC-MUSIC algorithm are shown in Figure 14a,b. It can be seen that the MUSIC algorithm does not converge, and the STC-MUSIC algorithm can estimate the direction of the interference signal normally, there are two distinct peaks in Figure 14b. It should be noted that the elevation and azimuth angles in Figure 14b are meaningless because the directional pattern of the array antenna is not calibrated with the actual orientation accurately, including the calibration and measurement of the array element coordinates and the angle of arrival of the interference.

## 5. Conclusions

For the DOA estimation of the interference signal by GNSS receivers, the STC-MUSIC algorithm is proposed in this paper. By effectively extending the order of the covariance matrix and introducing the focusing parameter, the accuracy and robustness of the DOA estimation for the interference signal are significantly improved. Meanwhile, a method of variable step size peak search is proposed to reduce the amount of calculation of the STC-MUSIC algorithm. The effectiveness and advantages of the STC-MUSIC algorithm are verified and evaluated via simulation and experiment. In particular, for multi-interference situations, the algorithm compensates for the shortcomings of the traditional MUSIC algorithm effectively.

## Figures and Tables

**Table 1:** Basic simulation parameters.

Category	Parameter	Value
Signal parameter	Sampling frequency	62 MHz
	RF frequency	1268.52 MHz
	Intermediate frequency	46.52 MHz
Array parameter	Number of array element	4
	Array type	Circular array
	Element spacing	Half wavelength

**Table 2:** Interference scene parameters.

Scene 1	Value
Number of interference	1
Type of interference	BPSK
Interference bandwidth	2.046 MHz
JNR(Jamming-to-noise ratio)	0 dB
Scene 2	Value
Number of interference	2
Type of interference	a BPSK and a Gaussian
Interference bandwidth	2.046 MHz
JNR	0 dB

**Table 3:** Interference parameters.

Parameter	Value
Number of interference	2
Type of interference	Gaussian
Interference bandwidth	4 MHz
JNR	20 dB
